# Bacteriophage Adsorption: Likelihood of Virion Encounter with Bacteria and Other Factors Affecting Rates

**DOI:** 10.3390/antibiotics12040723

**Published:** 2023-04-07

**Authors:** Stephen Tobias Abedon

**Affiliations:** Department of Microbiology, The Ohio State University, Mansfield, OH 44906, USA; abedon.1@osu.edu

**Keywords:** adsorption rate constant, bacteriophage therapy, biocontrol, biofilm, mass action, phage–antibiotic synergy, sorptive scavenging

## Abstract

For ideal gasses, the likelihood of collision of two molecules is a function of concentrations as well as environmental factors such as temperature. This too is the case for particles diffusing within liquids. Two such particles are bacteria and their viruses, the latter called bacteriophages or phages. Here, I review the basic process of predicting the likelihoods of phage collision with bacteria. This is a key step governing rates of phage-virion adsorption to their bacterial hosts, thereby underlying a large fraction of the potential for a given phage concentration to affect a susceptible bacterial population. Understanding what can influence those rates is very relevant to appreciating both phage ecology and the phage therapy of bacterial infections, i.e., where phages are used to augment or replace antibiotics; so too adsorption rates are highly important for predicting the potential for phage-mediated biological control of environmental bacteria. Particularly emphasized here, however, are numerous complications on phage adsorption rates beyond as dictated by the ideals of standard adsorption theory. These include movements other than due to diffusion, various hindrances to diffusive movement, and the influence of assorted heterogeneities. Considered chiefly are the biological consequences of these various phenomena rather than their mathematical underpinnings.

## 1. Introduction

“…the only properties of a spherical particle that are important are its diameter and speed.” [1], p. 104.

Phage therapy, or more generally phage-mediated biological control of bacteria, is the use of bacterial viruses to reduce numbers of pathogenic or at least nuisance bacteria as found within patients or environments [2,3,4]. This technique is based on the idea that adsorption of especially strictly lytic phages to susceptible bacteria can result in the death of those bacteria. These phages in turn are considered to display single-hit killing kinetics [5], i.e., from d’Herelle [6], p. 253, “…the bacteriophage is composed of particles, of which only a single one is liable to effect lysis…”. This is rather than requiring exposure of individual bacteria to multiple antibacterial units to effect strong antibacterial activity. Phage adsorption, as representing the initial ‘hit’ (attachment) required to commence phage-mediated bacterial killing, nevertheless still must occur for phage therapy to be effective, and ideally this adsorption will occur sooner rather than later [7].

The rate at which phage virions can adsorb targeted bacteria is a key factor in determining the timeliness of bacteria killing. This rate traditionally has been predictable, at least under simplified conditions, from knowledge of phage, bacterial, and environmental properties. In particular, a phage adsorption rate constant is derivable from phage virion diffusion rates, bacterial size, and the likelihood of irreversible virion attachment following encounter with a bacterium [8]. Phage adsorption rate constants also, as well as more commonly, may be measured empirically. The latter involves determinations of the rapidity of exponential declines in the number of free phages within bacterial cultures, or instead measurements of increases in numbers of phage-infected bacteria. For either case, these determinations are made particularly under conditions where both phage and bacterial replication is insubstantial [8,9,10,11,12].

The greater a phage’s adsorption rate constant, then the faster that individual phages are able to acquire bacteria; thereby the faster that bacteria may be killed in the course of phage therapy. This rate is dependent not just on a phage’s adsorption rate constant, however, but also, per the law of mass action, on the concentration of free phages within an environment (phage in situ titers) as well as concentrations of adsorbable bacteria. From knowledge of phage adsorption rate constants, and particularly also of in situ phage titers, a great deal of phage therapy pharmacodynamics thereby may be predicted, i.e., the potential for a given concentration of phages to bring an infecting bacterial population under control [7]. As a consequence, as I have argued elsewhere, it is crucial, to the extent possible, that at least initial in situ phage titers be reported when describing phage therapy experiments [13].

**Figure 1:**
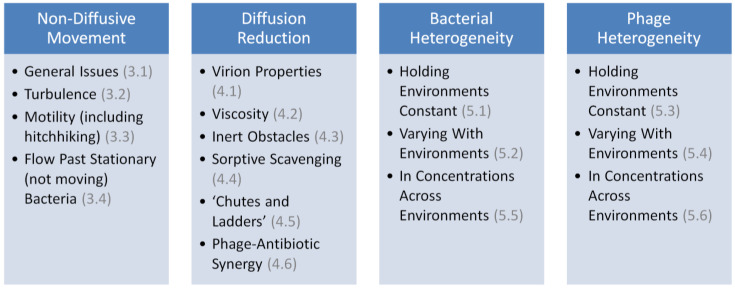
Summary of topics covered. Section numbers are indicated parenthetically, in gray. Not displayed in this summary is Section 2, which considers some theory of phage adsorption, as is considered in additional detail also in Appendix A.

Considered here is a look at the basic underlying theory of phage adsorption rates as well as, and especially, numerous potential complications on actual rates of phage adsorption, particularly as those complications may have biological relevance. We begin with consideration of the place of adsorption in the phage life cycle and then indicate the role of the phage adsorption rate constant in determining the rapidity of aspects of that life cycle. Then described are how non-diffusive movements can impact rates of phage adsorption along with how considerations of diffusion alone can deviate from the expectations of basic theory. Lastly covered are how multiple heterogeneities (environmental, physiological, in terms of concentrations) can affect rates with which phages adsorb bacteria. See Figure 1 for further summary of what is presented.

## 2. Phage Adsorption within the Phage Life Cycle

**Figure 2:**
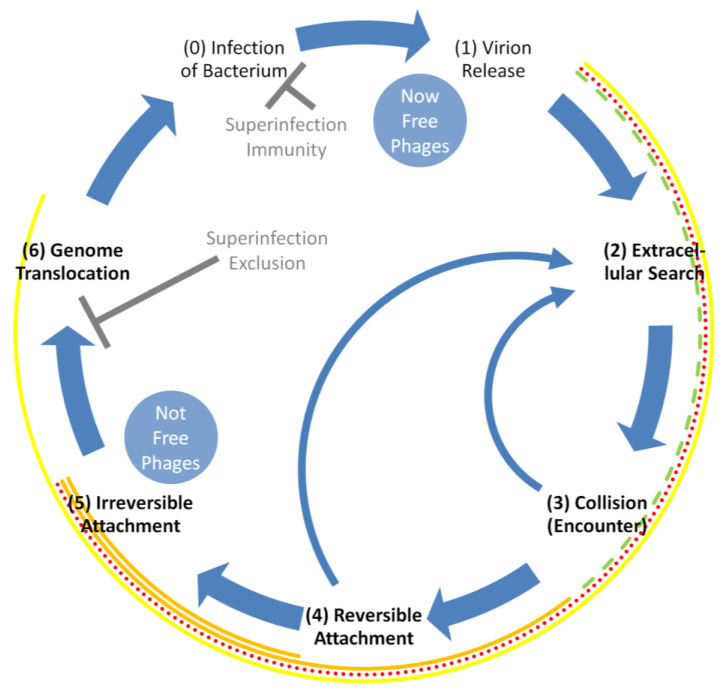
Life cycle of a bacteriophage with emphasis on phage adsorption. At an upper limit, adsorption involves steps 2 through 6. (1) is from a phage-infected bacterium. (2) is for new bacteria to infect. (3) is of a virion with a bacterium. (4) can be followed either by irreversible attachment to a bacterium’s surface (5) or instead a return to the extracellular search (as too can collision be followed by return to the extracellular search if reversible attachment fails to occur; both of these return processes are shown as narrow, curved arrows). (6) is into a bacterium’s cytoplasm. Superinfection exclusion [14,15,16], if present (first inhibition arc, “⊥”, middle-left), can serve as a block on this genome translocation. Though not otherwise considered here, contrast superinfection exclusion with superinfection immunity, where the latter represents instead a type of post-translocation block on the progression of a phage infection [17] (second inhibition arc, top-center). The surrounding, multicolored arcs represent different perspectives on how the processes of adsorption might be described. These range from just irreversible attachment (innermost, orange arc) to a combination of reversible and irreversible attachment (second-innermost, orange arc) to the extracellular search through irreversible attachment (third-innermost arc; shown as both red and dotted) to the extracellular search through phage genome translocation (outermost yellow arc). In terms of adsorption rates, it is as represented by the dotted-red arc that is relevant, though much of the consideration here is on just step (2) as ending in step (3) (dashed-green arc).

Phage adsorption is a complex, multi-step process that begins, arguably, as early as the point of virion release from a phage-infected bacterium and ends (again, at least arguably) as late as the point of phage genome translocation into the now phage-infected bacterium (Figure 2). That is, the phage life cycle, with emphasis on adsorption, takes place, in order, like this:

(0) Infection →
(1) Release (of virions from a cell) →
(2) Movement (of virions; a.k.a., ‘extracellular search’ or ‘transport’ [1]) →
(3) Collision (a.k.a., encounter, of virions with a potentially adsorbable bacterium) →
(4) Reversible attachment (of a virion to a cell’s surface [8]) →
(5) Irreversible attachment (of a virion to a cell’s surface) →
(6) Translocation (of a phage genome into a bacterium’s cytoplasm) →
(0) Infection.

At a maximum, all but infection (0) and release (1) are explicitly associated with the adsorption process. Reversible and irreversible attachment can also be described as reversible or irreversible *adsorption*. See Table 1 for adsorption-related definitions.

Note that collision (3) or reversible virion attachment (4) to a bacterium’s surface can instead be followed by a return to the extracellular search (2). These are shown as narrow, curved arrows in Figure 2, indicating that irreversible attachment (5) has at least temporarily failed to occur. In addition, given the display of superinfection exclusion by the adsorbed bacterium [14,15,16], then irreversible attachment (5) is not necessarily followed by successful phage genome translocation but instead the phage life cycle is terminated prior to infection (with infection defined here as beginning with phage genome entrance into the bacterial cytoplasm). Adsorption, even given the occurrence of superinfection exclusion, however usually still will have been said to have occurred, thereby still contributing to what Koch [18] refers to as an ‘encounter efficiency’ and Murray and Jackson [1] refer to as an ‘effective contact rate’. See, e.g., [19,20,21] for reviews of more molecular aspects of phage adsorption as well as [22] for a review of resulting phage genome translocation.

### 2.1. More-Truncated Descriptions of Phage Adsorption

Contrasting the above considerations, Stent [8], p. 88, stated, “As a consequence of these collisions the phages become fixed, or adsorbed, to the cell surfaces”. Adsorption from that perspective thus consists at the most of steps (4) and (5) (Figure 2), which of course is somewhat less expansive than steps (2) through (6) as outlined above. Stent’s description makes sense, however, as an adsorbed phage is a virion that has attached. Furthermore, all previous steps as involving still-free phages are just leading up to this attachment. Slightly less truncated, the process of phage adsorption instead can be viewed as synonymous with the virion extracellular search (2), which is followed by virion collision with a susceptible bacterium (3) and then, after reversible attachment (4), permanent phage attachment (5): movement (2) → collision (3) → reversible attachment (4) → irreversible attachment (5). That is the perspective emphasized here.

Phage adsorption rates from this latter viewpoint are predominantly functions of virion diffusion rates (2) along with host size as determining virion collision rates (3). Also relevant is the likelihood of reversible attachment given collision (4), and then, the likelihood of virion irreversible attachment given reversible attachment (5). These latter two steps are not given a substantial emphasis here, however, because in practice, distinguishing between steps (3) and (4) may be ignored [23]. The duration of the transition from reversible to irreversible attachment also can be fast relative to the duration of the extracellular search, particularly given lower bacterial concentrations. What cannot be ignored, though, is the likelihood of irreversible virion attachment given collision, which will be dependent on a combination of phage affinity for adsorption receptor molecules found on a bacterium’s surface under ideal conditions (next section), the number of those molecules, i.e., their density as found on bacterial surfaces (Section 5.1), and the extent to which conditions for reversible or irreversible adsorption are not ideal, such as due to too low or two high adsorption factor concentrations, pH, osmolarity, or temperatures (Section 5.4). 

**Figure 3:**
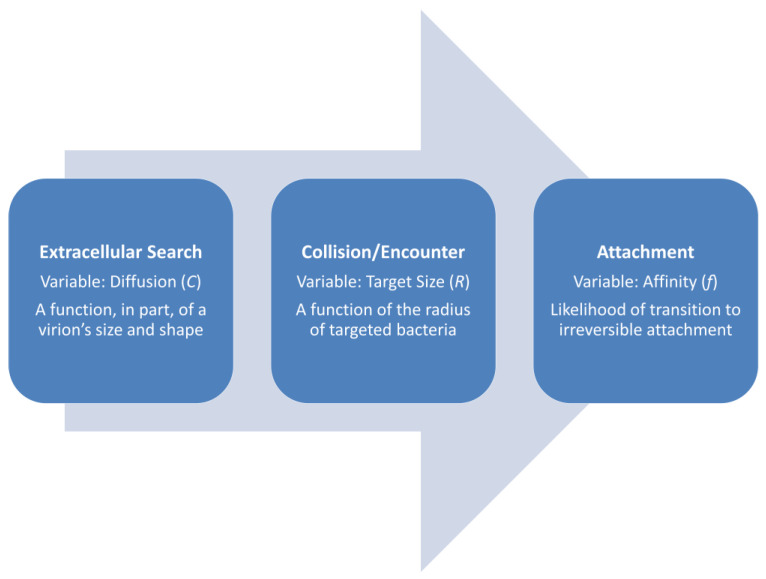
Factors impacting phage adsorption rates, diffusion through irreversible attachment. Variables are as introduced in Section 2.2 and Table 1. Together, they serve to determine a phage’s adsorption rate constant (*k*) for a given bacterium under a specific set of conditions.

Overall, then, adsorption rates can be determined as the time required to proceed from step (2) to step (5), starting with a free phage and ending with an irreversibly attached, i.e., adsorbed virion. Generally, however, the focus here is predominantly on how much time it takes for the transition from step (2) to step (3) to occur (equivalently: “Quantity *D* [for diffusion constant] is the only one related to the time scale.” [24], p. 138, as translated from German), and then the likelihood of subsequent irreversible adsorption (5). Virion adsorption rates, as defining adsorption rate constants, are therefore expected to be greater given faster virion diffusion, greater host cell size, and higher virion attachment affinities for a target host (Figure 3). These three factors in phage biology traditionally are collapsed into a single parameter, i.e., the noted phage adsorption rate constant.

Adsorption rates can also be affected by non-diffusive forms of motion occurring between phages and bacteria, particularly if these are fast in comparison to rates of virion diffusion (Section 3). Movement via diffusion itself also can be modified by various circumstances (Section 4). Both likelihoods and rates of virion adsorption in addition can vary for reasons other than due to individual rates of virion motion (Section 5).

### 2.2. The Phage Adsorption Rate Constant

Many of the especially non-molecular subtleties associated with phage adsorption can be explored by considering the phage adsorption rate constant, *k* [8,25]. This is a special case of a more general variable that can be described as a “collision kernel” or a “collision frequency”, with units of volume^−1^ time^−1^ [26] and as attributed to Smoluchowski [24]. The collision kernel thus describes the “number of collisions per unit volume and time” [26] (p. 720), and there are three aspects to it: particle motion, particle size, and what might be described as a collision efficiency [26].

For phage adsorption, there is a tendency to place limits on these factors when considering the theoretical values of *k*. These include:

1. To limit descriptions of particle motion to just that of virion diffusion (that is, assuming that hosts are comparably stationary and that viruses only move as a consequence of diffusion; see Section 3 for exceptions to the latter). Diffusion here is abbreviated as *C*, after Stent [8], for the diffusion *C*onstant.
2. To limit considerations of particle size to just that of host cells, assuming that virions are small relative to the size of bacteria, though note that ‘jumbo’ phages do exist for which that size discrepancy is not as great [27].
3. To invoke considerations of collision efficiency to describe the likelihood of virion attachment to a bacterium given encounter with that bacterium. The latter here is abbreviated as *f*, also after Stent, which is for e*f*ficiency. This particularly is the efficiency of the transition from step (3) to step (5) as considered above (Figure 2).

Thus, from Stent [8], as based on Smoluchowski [24], we have

(1) k=4πRCf,

where *R* is the radius of an idealized, spherical, target bacterium. The order of these variables in the equation also is as provided by Stent [8]. In words, the adsorption rate constant is a function of bacterial size (varying by *R*) × how fast a virion diffuses (*C*) × the likelihood of adsorption given collision (*f*). Thus, though in a different order from as presented in the equation: virions move (*C*), they encounter bacteria (differing in size by *R*), and then they may or may not ‘stick’ (*f*) (Figure 3).

The parameter *C* will vary with virion properties, such as virion size (where large particles tend to diffuse more slowly than smaller particles; Section 4.1). *C* also will vary with environment properties, such as the viscosity of the medium through which diffusion is occurring (Section 4.2). The efficiency parameter, *f*, on the other hand, will tend to vary as a function of both phage and bacterial properties as well as environmental parameters such as solute concentrations (for the latter, see Section 5.4). Stronger affinity by virions for the phage receptor molecules found on a bacterium‘s surface [28,29] and greater numbers of those receptor molecules, also as found on a bacterium‘s surface (Section 5.1), will for example tend to increase *f* towards a maximum value of 1.0. If *f* equals 1.0, then every phage encounter with a bacterium results in phage irreversible attachment, though *f* is thought to typically be at least a tiny bit less than 1.0. A value for *f* of zero, on the other hand, would imply that no phage–bacterial collisions result in irreversible virion attachment. That is, given the latter, then phage adsorption does not occur even if collisions do occur.

### 2.3. Adsorption Rate Generalizations

In review, we expect faster phage adsorption given (i) greater bacterial size (though potentially affected also by bacterial shape), (ii) faster virion movement (though for determining adsorption rate constants, this movement should be limited to diffusion), (iii) higher virion affinity for individual cell-surface receptor molecules, (iv) adequate numbers of receptor molecules on bacterial surfaces, and (v) substantial bacterium movement, vs. just phage movement, or, for that matter, fluid flow over otherwise motionless bacteria (Section 3). We can in addition consider the consequences of bacterial clustering—such as bacteria ‘clumping’ into microcolonies—on the likelihood of virion encounter with bacteria following virion release from phage-infected bacteria. Rather than explicitly impacting adsorption rate constants, however, the latter instead is more a function of a local concentrating of bacteria affecting phage adsorption rates (Section 5.5), an issue that we begin to touch upon also in the following paragraph.

**Figure 4:**
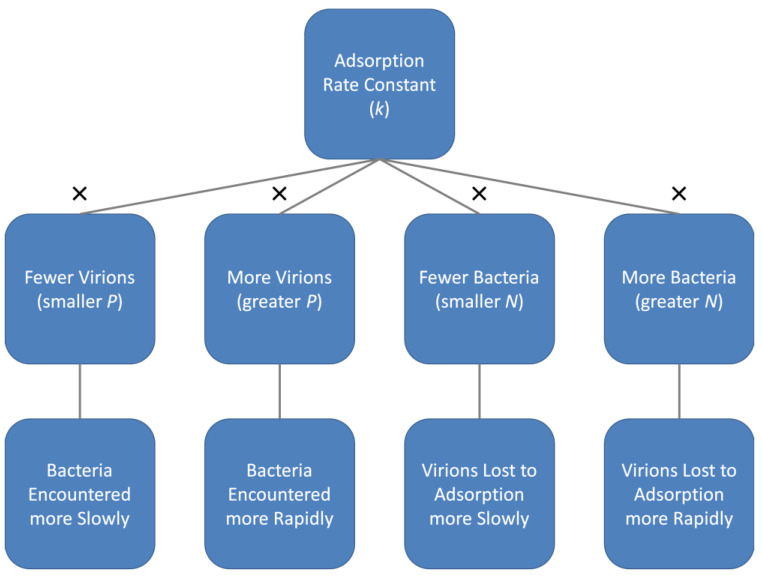
Impact of phage and bacterial concentrations on adsorption rates. “Virions” are as found extracellularly, i.e., as “Free phages”, and “Bacteria” are phage-adsorption susceptible. Variable names are abbreviated as indicated parenthetically and “×” refers to the multiplication sign, i.e., as resulting in *k* × *P* or *k* × *N*. See Appendix A for exploration of the underpinnings of the presented assertions including a visualization of equivalent concepts of free phage and bacterial half-lives as functions of bacterial (*N*) or virion (*P*) densities in environments.

A slightly different perspective on phage adsorption rate constants is that they represent the likelihood that one phage will irreversibly attach to one bacterium, each suspended in a given volume of medium over a given span of time. This, however, is not the same as the rate that a phage will adsorb within a given environment nor the rate at which a bacterium will be adsorbed, and this is because both phage and bacterial concentrations can range over more than just single individuals per the volume in question. Thus, phage adsorption rates will vary as a function of not just the magnitude of a phage’s adsorption rate constant but also will vary with phage and bacterial concentrations (Figure 4).

### 2.4. Phage Adsorption Rates

Degrees of phage population adsorption to a bacterial population can, at least in theory, be a primary measure of phage therapy success [7] and are a function of at least four parameters. These are phage titer, i.e., phage concentration (*P*), bacterial concentration (*N*), the adsorption rate constant (*k*), and time (*t*). The adsorption rate constant, as described in the previous section, defines the likelihood of a single phage adsorbing a single bacterium within some unit volume (generally 1 mL) over some unit of time. A typical unit of time is 1 min, but for many authors 1 h is used instead, and per second is seen as well [30]. It therefore is important to keep track of these units when comparing adsorption rate constants or, instead, when considering any issues that are based on the magnitude of these constants. Specifically, the number of virions that are expected to adsorb over 1 h can be as much as 60 times that over 1 min. For modeling purposes, *k* as based on 60-min time units is *always* 60 times larger than *k* as based on 1-min time units. As real-world adsorption rates involve more than just adsorption rate constants, such as depending on phage titers as well, there is a potential for more adsorptions occurring in a given 1 min period than per min over 60 such minutes, such as resulting from declines in numbers of free phages after that first minute due to phage adsorption (Appendix A.4.2).

Regardless of what units are used to define *k*, this phage adsorption rate constant serves as the basis for understanding how fast a given phage is able to adsorb a given bacterial strain. That statement comes with a caveat, though, that circumstances can modify actual adsorption rates, as is the consideration of the rest of this review. These include as functions of phage or bacterial concentrations (Appendix A, and as summarized in Figure 4), due to various forms of non-diffusive movement (Section 3), because of modifications of diffusive movement (Section 4), or instead as associated with inhomogeneities found within the adsorption milieu (Section 5). See Storms and Sauvageau [25] for a complementary perspective especially on modeling of phage adsorption.

### 2.5. Importance of Different Variables and Parameters

The general question being addressed here is, what influences phage adsorption rates, with particular emphasis on rates of phage encounter with bacteria. In Appendix A, as summarized in Figure 4, we consider the importance of phage and bacterial concentrations in determining those rates, indicating that bacterial concentrations (*N*) are important for determining rates that free phages are lost to adsorption whereas phage concentrations (as free phages; *P*) are important toward determining rates that unadsorbed bacteria are lost to phage adsorption. An additional consideration is the importance of *k*, the phage adsorption rate constant. In terms of rates that free phages are lost to adsorption, *k* is particularly relevant at low bacterial concentrations. This especially can be envisaged with respect to the affinity aspect of *k*, i.e., abbreviated as *f*. 

When bacterial concentrations are high enough, then time between phage collisions with bacteria becomes small. As a result, failure of reversible adsorption to be followed by irreversible adsorption can be quickly followed with new attempts at adsorption, such as involving different bacteria found in the same volume. Thus, for example, if only one in ten phage encounters with bacteria result in irreversible adsorptions (thus, *f* = 0.1), then on average it will take 10 s for the latter to occur, assuming only 1 s between collisions, e.g., as may be seen at higher bacterial concentrations. If we reduce those concentrations ten-fold, however, then the average time between collisions instead will be 10 s, and therefore, it will take on average 100 s until a virion irreversibly adsorbs. An additional issue, however, is that the transition from collision to irreversible adsorption can become slow relative to rates of collision as bacterial concentrations become very high [31].

Equivalent arguments can be made with regard to the importance of *k* in determining how rapidly unadsorbed bacteria become adsorbed. At higher phage densities, that duration can be relatively short even if *f* is somewhat smaller than 1. However, if phage concentrations decrease by ten-fold, then that average rate will be ten-fold lower, and so on. Another way of saying this is that low phage affinities for bacteria, as resulting in smaller values of *k*, can be compensated for by starting with higher in situ phage titers. Indeed, that would be the case regardless of why *k* is smaller, as generally speaking starting with, e.g., ten-fold more phages, as titers, will result in ten-fold faster loss of bacteria to phage adsorption. This is at least so long as *k* is greater than zero, and adsorption environments are well mixed. 

In terms of example values, on average, one-second intervals between virion collisions for an adsorbing phage will occur given *N* = 4 × 10^8^ bacteria/mL = 1/*k*, with *k* = 2.5 × 10^−9^ mL^−1^ min^−1^ [8]. An average time between collisions of 10 s instead would be seen with a bacterial concentration of 4 × 10^7^ per mL, and so on. Similarly, a bacterium should experience a collision with a phage every second given *P* = 4 × 10^8^ free phages/mL, or every ten seconds for *P* = 4 × 10^7^ free phages/mL. This though would be slower with smaller values of *k*, e.g., on average every ten seconds, given *P* = 4 × 10^8^ free phages/mL but with *k* equal to 2.5 × 10^−10^ instead of *k* = 2.5 × 10^−9^, or faster with larger values of *k*, etc. 

Moreover, in general faster virion movement (Section 3) can have a consequence of increasing the effective value of *k*. This is by making the diffusion component of *k* (*C*) smaller than the actual rates that phages are able to explore the volumes they occupy, toward colliding with susceptible bacteria. Alternatively, simply slower virion diffusion (Section 4) will result in smaller values of *k*, decreasing the rate that phages are able to explore those volumes.

## 3. Non-Diffusive Movement

For movement, or “Motion” [1], to be relevant to phage adsorption to bacteria, then that movement must be relative. Thus, diffusion randomly can bring phage virions into contact with bacteria, though alternatively diffusion can, and will, move virions away from bacteria. Alternatively, fluid flow alone, in the form of laminar flow, can in principle carry particles parallel to each other, as too can bulk movement of a sampling of an environment from location to another. Other forms of movement besides diffusion also can move virions and bacteria relative to each other. These other forms of motion most notably [32] include turbulent movement (Section 3.2), bacterial motility (Section 3.3), and the flow of virus-containing fluids over stationary bacteria (Section 3.4). First considered, in Section 3.1, are possible general impacts of non-diffusive, relative movements on phage adsorption rates.

### 3.1. Anticipated Impact of Relative Movement (Theory)

Murray and Jackson [1] considered, theoretically, the effect of movement or motion, besides that associated with particle diffusion, on virus adsorption rates. An overall conclusion was that while greater velocities should increase adsorption rates (or at least virion-encounter rates), increases in rates of motion over those associated with diffusion alone should be more relevant for moving particles that possess larger diameters, such as the size of protists, than for particles with smaller diameters, such as bacteria. Curiously, the primary comparison made by Murray and Jackson in reaching the latter conclusion involved not changes in encounter rates as a function of absolute increases in rates of cell movement. Instead, modifications in encounter rates were made as a function of changes in speeds as expressed in units of diameters of idealized spherical cells traveled per unit time—the problem here not being the assumption that cells are spherical but instead that encounter rates in terms of absolute increases in cell velocities was not what was being compared.

Thus, from [1], an increase in rates of movement from 0 to 1250 μm/s was predicted by Murray and Jackson to increase encounter rates by ten-fold for a 125 μm diameter, and thereby *large* cell, whereas an increase from 0 to 50 μm/s was predicted to have only a negligible impact on virion encounter rates for a 1 μm diameter cell. However, the velocity, 1250 μm/s, is 25 times faster than 50 μm/s. Therefore, a 50 μm/s velocity should have much less of an impact on encounter rates for the larger cell (as equivalent to 0.4 cell diameters/s) relative to the velocity of 10 cell diameters per second also for the larger cell (=1250 μm/s). Indeed, Murray and Jackson report that for a cell of diameter of 125 μm, a velocity of 1 cell diameter per second increases encounter rates by “several fold”, but I note here that this 125 μm/s is still 2.5-fold faster than 50 μm/s.

This is not to say that the general conclusion from Murray and Jackson is necessarily incorrect, that a large cell in comparison with a very small cell will experience a greater influence of relative cell movement on virion encounter rates. Furthermore, larger cells such as protists may be able to swim faster than smaller cells. It is just that as measured in absolute velocities, relative changes in encounter rates with increased speeds can be much lower than when velocities are described instead in terms of cell diameters. Furthermore, as cell diameters increase from 1 μm, such as to 5 μm, the impact of cell movement becomes less negligible, with roughly a doubling in encounter rates going from 0 to 50 μm/s (see Murray and Jackson’s Figure 2 for additional summary of these effects; also see Section 3.3). Therefore, we can at least tentatively anticipate that, while for very small bacteria, substantial cell movement relative to the position of viruses may have little effect on increasing rates of virion encounter, somewhat greater increases in adsorption rates might be seen with the movement of larger bacteria relative to virions, or the movement of virions relative also to larger adsorption targets (Section 3.4).

A corollary may be that, since virions are especially small in diameter, there would be minimal utility for a hypothetical virus to display motility toward enhancing their adsorption rates, at least within an environment in which bacteria are both plentiful and homogeneously distributed, that is, so that virion diffusion alone can provide somewhat rapid encounter rates (Section 2.5). This proposed low utility to virion motility, though, is only so long as random virion movement over substantial distances would provide only minimal utility toward increasing phage adsorption likelihoods. Given heterogeneous bacterial distributions within environments (Section 5.5), or simply low overall bacterial densities, i.e., where relatively long distances must be traversed for phages to reach new pockets of higher bacterial densities, then phage motility might instead be useful, were that actually possible. See Section 3.3 for possible work arounds for this problem of supplying virions with motility, as could be useful especially for phages dealing with heterogeneous bacterial distributions.

Similarly, it is important to keep in mind that in terms of the rates that bacteria are adsorbed by phages, increasing the speed of movement of cells should be less relevant at higher vs. lower phage concentrations (Section 2.5). This is just as greater phage velocities over that which can be achieved via diffusion alone would be less relevant, toward increasing phage rates of finding new bacteria to infect, the greater bacterial concentrations.

### 3.2. Turbulence

Turbulent movement is the jostling of particles randomly but at speeds that are higher than those associated with diffusion. This has the effect of mixing environments, thereby creating greater homogeneities in concentrations, but also can serve to increase the rates at which virions randomly collide with bacteria over that of diffusion alone. The resulting effect can be small, however, unless mixing is occurring in a manner that “is effective at the microscopic level” [18], p. 316. Koch [18] considers just such a scenario involving fluid movement though capillary tubes, which he suggests could result in increases in phage adsorption rates of 4.5- to 14-fold. 

Levels of turbulence nevertheless can instead be excessive to the point of disrupting rather than enhancing adsorption, i.e., by interfering with the transition from virion encounter to irreversible adsorption. This can be seen while attempting to adsorb phage T4 virions within a Waring blender [33], i.e., as also was used by Hershey and Chase to shear off even fully adsorption virions [34]. This approach, however, presumably could be used as part of a process of rapidly ceasing adsorption if that were desired, since otherwise such excessive jostling is thought to have little impact on either free phage or bacterium.

### 3.3. Motility

Motility, particularly as flagella driven [35], is not universal among bacteria, either phylogenetically or circumstantially [36]. Resulting increases in rates of cellular movement relative to diffusing virions [9] nevertheless can be particularly relevant the faster the movement as well as the larger the size of the moving entity (Section 3.2). It has been argued, though, that this movement actually is less relevant for comparatively small bacteria vs. the movement of, e.g., single-celled eukaryotes (Section 3.1), though still likely is not completely irrelevant in terms of increasing rates of phage adsorption. Both Koch [18] and Berg and Purcell [37] thus proposed a possible doubling of adsorption rates given typical bacterial motility, as too is suggested by the calculations of Murray and Jackson ([1]; Section 3.1). Koch [18] furthermore suggested that *Vibrio cholerae* moving at a substantial speed of 50 times that of its diameter per second could give rise to a roughly four-fold increase in phage encounter rates (“320% greater”) relative to an equivalent stationary bacterium of the same size. 

Potentially further reducing the impact of motility is that bacilli moving predominantly with their smallest cross-sections leading should display less of an increase in encounter rates with phages, as due to that motility, than their overall size might suggest. This would be relative to either turbulent movement or movement, e.g., such as tumbling, that instead sweeps their breadth through phage-containing environments, and particularly so the greater the cell length of the tumbling bacterium. It could also be relevant, however, to explore the extent to which virion encounter with the sides of a motile bacterium might impact likelihoods of subsequent irreversible attachment, i.e., as possibly affecting the magnitude of *f*. That is, is it possible that bacteria, while running, even if more likely to encounter virions due to greater rates of relative movement, they still are less likely to be adsorbed per encountered virion along their flanks than a stationary bacterium of the same size and shape due, perhaps, to shear effected by that movement? 

To the extent bacteria move about as streptobacilli, then there would be even less bacterial surface leading through environments relative to the collective adsorptive area of these cells. Alternatively, such chains of bacteria will together be more susceptible to collisions with virions as resulting from diffusive or turbulent movements than the same individual bacteria if separated or streptobacilli consisting of shorter chains. Any of these encounters could then lead to cell-to-cell virion reproductive propagation through these bacterial chains (Section 5.5). 

Thus, (i) longer bacteria may have a lower fraction of their leading surfaces exposed to virions but still possess greater surface areas overall than shorter cells, (ii) bacteria by moving faster might be able to lower per-collision phage adsorptions rates (speculation, i.e., as due to shear forces) though should overall be colliding with more phages at least at the leading portion of cells, and (iii) for motile filaments—by being separated into multiple cells rather than existing as individual, very long cells—then each individual cell should have a lower likelihood of encountering a phage (due to reductions in surface area), but still that might not be enough to protect overall multi-celled filaments from phage-mediated exploitation. A key assumption in these considerations nonetheless is that cellular movement is occurring through a virus-containing locale rather than that cells instead are moving away from phages [38,39]. Motile bacteria, however, instead might inadvertently move toward greater concentrations of otherwise stationary or only locally diffusing phages, ones to which they are susceptible. Thus, inspired as a mechanism by the work of Barr et al. [40,41], I have described phages as potentially serving as sit-and-wait or ambush predators of bacteria, that is, with bacteria moving toward more stationary virions rather than virions diffusing toward more stationary bacteria [42,43].

Another issue, not directly related to adsorption rates but nonetheless involving bacterial motility is that of hitchhiking. In this case, rather than bacteria moving toward free phages, or away from them, instead the bacteria themselves are carrying phages, as phage infections [39,44]. Indeed, this could represent an advantage for phages able to display lysogenic cycles in that this would allow phage carriage, while infecting bacteria, over greater spans of time and thereby over greater distances [45]. Phages also are capable of hitching rides on non-bacterial organisms, e.g., such as invertebrates [46,47]. Possible as well is phage movement facilitated by reversible attachment to motile but non-host bacteria [48], including with those non-host bacteria then moving along fungal mycelia [49,50].

### 3.4. Flow Past Stationary (Not-Moving) Bacteria

Also of relevance, particularly to phage ecology, is the movement of virions via fluid flow relative to immobile or less mobile bacteria. Such flow, even if relatively slow, e.g., 1 mm/s = 1000 μm/s [1], can be quite rapid in comparison to rates of bacterial movement that are due to cell motility alone, e.g., 50 μm/s (Section 3.1). This in principle should increase the likelihood that bacterial biofilms, microcolonies, or individual cells as adhered to surfaces will encounter a virus (also Section 3.1). Such flow relative to clusters of bacteria might in fact be of particular relevance to phage adsorption, as overall diameters of these clusters or clumps (of microcolonies or biofilms) can be much larger than those of individual bacteria [51] (see also Section 5.5). This latter point, however, needs to be tempered by considerations of to what degree a given clump of bacterial cells is exposed to flow, such as due to only a fraction of, e.g., a microcolony’s surface not being protected from flow by other materials. Those other materials could include other, unrelated bacterial microcolonies and/or other bacteria found within the same mixed-species biofilm (see somewhat equivalently, Section 4.3).

Given larger volumes of flowing water but relatively few phage-infected bacteria supplying virions to that water, e.g., such as for a stream flowing over biofilm-encrusted rocks, then fewer phages may be present within those larger volumes relative to more static environments. In particular, the latter (little or no flow) might allow virions to accumulate in the vicinity of biofilms (Section 5.6). Fluid flow therefore not only may increase rates of virion encounter with stationary bacteria, when phage concentrations across environments are homogeneous (Section 3.1), but so too fluid flow could more rapidly move virions away from clusters of those same bacteria, i.e., given ongoing virion replication in a biofilm’s vicinity. Note in any case that for phage therapy, rather than providing a continuous flow of phage-containing fluid, an easier means of increasing likelihoods that bacteria will be encountered by virions can be to provide phages at higher titers (compare, for instance, Table A1, Table A2, Table A3 and Table A4).

## 4. Reductions in Rates of Virion Diffusion

There are a number of ways that reliance on diffusion for movement can result in either slower or less adsorption. This is not relative to movement by non-diffusive means but instead is associated with a dependence on just diffusion for adsorption. In particular, rates of diffusion will vary with virion properties (Section 4.1), with the viscosity of fluids (Section 4.2), and to the extent that inert partial barriers to movement exist (Section 4.3). In addition is the issue that infecting phages are not diffusing phages (Section 4.4), though on the other hand, in certain circumstances intracellular movement could, I speculate, be more rapid than extracellular movement (Section 4.5). The latter I associate possibly with aspects of a concept described as phage–antibiotic synergy (Section 4.6). Diffusion, it should be noted, is not in any case necessarily a rapid form of particle movement even absent the various reductions described here. Saltzman [52] in fact has estimated that it would require 800 years for a molecule of albumin, a kind of protein that is much smaller than a virion particle, to migrate just two meters via diffusion alone.

### 4.1. Virion Properties

Key virion properties impacting rates of diffusion are their size and shape. Specifically, the larger the virion, or substance generally [1], then the slower their diffusion (with diffusion rates an inverse function of particle radius according to the Stokes-Einstein equation [53]). This property is equivalent to the reason that we disregarded bacterial diffusion relative to that of phage diffusion in predicting phage adsorption rate constants (Section 2.2), i.e., as bacteria tend to be that much larger than virions. That this slower diffusion would result in smaller phage plaques displayed by larger phage virions [54] also has long been speculated [55]. This, though, does come with a caveat that larger phages may display smaller plaques for reasons other than slower rates of virion diffusion. Indeed, Elford and Andrewes [55] pointed out, e.g., “the rate of its multiplication and other factors will certainly come into play as well” (p. 455). Subsequently [56], it became understood also that the T-even phage lysis inhibition phenomenon could cause smaller plaque sizes by these larger phage virions as well [15].

In addition are virion appendages, including tail fibers. These can further extend a virion’s size in ways that can create greater fluid dynamic drag. This can be observed in terms of sedimentation rates in the case of whether these tail fibers are or are not retracted [57,58], i.e., such as resulting in about a 1.5-fold larger sedimentation coefficient (indicating faster sedimentation) when tail fibers are more retracted, in phage T2 as seen at lower pHs [59]. Thus, smaller, simpler, indeed spherical virions are expected to diffuse faster than larger, more complexly shaped virions. All else held constant, the latter virions therefore should possess smaller adsorption rate constants and therefore slower adsorption.

### 4.2. Viscosity

The second issue regarding diffusive movement is the extent to which environments are more viscous than pure water. Greater viscosity has the effect of slowing diffusion, e.g., [53], while also, by definition, slowing fluid flow. An important question therefore is one of when it may be that a phage will be subject to environments possessing greater viscosities than pure water. One answer is to simply add solutes, which in most cases will increase a water solution’s viscosity as a function of solute concentration, though in some cases viscosity instead will be lowered [60]. Another answer is to lower temperate. For instance, while the viscosity of pure water is 0.69 milliPascal seconds (mPas) at 37 °C, it is 0.89 mPas at 25 °C, and 1.3 mPas at 10 °C [61] (diffusion rates, though, will also decline with temperature, independent of changes in viscosity, due simply to there being less kinetic energy present in lower-temperature systems). Another answer is blood plasma, which due to the presence of solutes as well as interactions between suspended proteins also has a higher viscosity than pure water, ranging from 1.1 to 1.3 mPas at 37 °C [62]. Phage adsorption rates thus will not necessarily remain constant but instead can change as a function of the properties of the aqueous solution in which adsorption is occurring, with adsorption rates varying in this case as a function of rates of diffusion rather than due to changes in either phage or bacterial properties.

Alternatively, various agents including agar and biofilm matrix can reduce the ability of environments to flow, thereby suggesting greater viscosity, but without necessarily inhibiting the diffusion (vs. movement via flow) of smaller substances. This can be particularly so for neutrally charged molecules that do not otherwise display weak chemical interactions with a thickening agent [63]. Nonetheless, even if weak chemical interactions are absent, such agents may still interfere with the diffusion of particles from point A to point B. That interference, however, is considered in the following section (Section 4.3). If something instead is attracted chemically to the thickening agent, or indeed any otherwise relatively stationary substance or entity, then this too can interfere with diffusion (Section 4.4), though not due to modifying the viscosity of the actual fluid within which diffusion otherwise would be occurring.

### 4.3. Inert Obstacles

Stationary objects can interfere with virion movement even if those objects, or materials, are inert relative to virion adsorptive affinities. Yin and McCaskill [64] for example argued that rates of phage diffusion can be slowed (“Hindered diffusion”) by the presence of bacteria to which virions cannot adsorb (“Diffusional barrier”, both p. 1642 [64]). The idea here is simply that these bacteria can interfere with phage diffusion to locations beyond those bacteria, i.e., by these adsorption-inert bacteria serving as obstacles. Phages in some cases also can reversibly adsorb non-host bacteria, though that is not the consideration in this section (see instead Section 4.4).

So too agar fibers [65], or biofilm matrix [66,67], can serve as otherwise inert obstacles to the diffusion especially of larger particles. This occurs without virions necessarily becoming permanently trapped—unless those virions are very large [54] or matrix concentrations sufficiently high—but instead makes random exploration by virions of their local environment less straightforward, therefore taking longer. It is possible as well that different regions of biofilm matrix may have different impacts on the magnitude of phage diffusion [68].

An interesting consequence of these considerations is that phage tails, as relatively thin virion appendages—perhaps particularly as seen with flexible-tailed siphoviruses—might exist as means of allowing for partial virion movement into bacterial biofilms, or glycocalyx more generally, that instead would block larger phage heads [54]. That is, with “Phage tails [serving] as polymeric substance probes” [69]. This actually is a fairly old idea, presented for example by Wilkinson in 1958 [70], though there with some skepticism. See too [71] for a more recent though still previous suggestion of this possibility. 

Perhaps related to these latter ideas is the reported “Explosive” ejection of antibacterial tailocins, i.e., bacteriocins consisting of phage tail-like structures lacking in virion heads, as generated by producing bacteria upon the latter’s lysis [72], as indeed could apply to whole phages as well. These tails perhaps may serve as physically more effective projectiles for targeting bacteria found in adjacent, biofilm matrix-encased competitor microcolonies than could instead whole phages with somewhat wider heads [73]. Specifically, tailocins may be able to not just more effectively reach neighboring, competing biofilms via their explosive discharge, but also might then be able to more effectively penetrate those biofilms than whole phage virions, i.e., due to the slimmer dimensions of phage tails relative to those of phage heads.

### 4.4. Sorptive Scavenging

A noted contrast to the impact of adsorptively inert obstacles on phage diffusion is when virions have affinity for environmental objects. This can include affinities for whole bacteria, for bacterial debris, or for membrane vesicles [74], with these affinities resulting in what can be described as sorptive scavenging [38]. In this case, not only can movement through that object be blocked, but the phage may also be delayed or outright prevented in its movement away from the blocking object or material. Much has been made of this idea for example in association with phage affinity for mucus, as can result in slower virion movement within mucus than is seen absent this affinity [40,41].

Perhaps of greatest relevance is simply phage adsorption to host bacteria, thereby derailing a virion’s diffusion [12], even if the now adsorbed phage remains viable intracellularly as a phage infection. Specifically, a phage infecting a bacterium is a phage that is not diffusing extracellularly, such as through agar or instead through biofilm matrix. This likely has an effect of slowing virion penetration into bacterial biofilms [12,38,75], but also explicitly has been implicated along with phage latent period length as an important factor in controlling the rate of growth of phage plaques. In particular, both longer phage latent periods (i.e., time not extracellularly diffusing) and greater adsorption affinity (increasing likelihood of a virion becoming not extracellularly diffusing) are thought to slow rates of plaque enlargement [64,75,76,77], which is a phenomenon that is driven by reaction- (i.e., infection-) diffusion mechanisms [64]. So too should simply greater concentrations of immobile, adsorbable bacteria serve as more effective even if only temporary barriers to further phage diffusion than fewer adsorbable bacteria [12].

### 4.5. Chutes and Ladders

What if a phage, or its biochemical underpinnings, can move faster inside of a bacterium than its virions can diffuse extracellularly? I dub this a potential ‘Chutes and Ladders’ mechanism. This is after the board game of the same name, which supplies a standard pathway of progression (as analogous to extracellular diffusion) along with means by which players may move more rapidly either backwards (chutes toward the start) or forward (ladders toward the finish). Here it is especially filamentous bacteria that could serve as both chutes and ladders. Thus, a phage that adsorbs to one end of such a bacterium may intracellularly progress and ultimately be released via lysis at or near the other end. During plaque development, this adsorption can occur nearer to the center of a plaque, with virion release thereby occurring into a plaque’s current periphery. If such intracellular movement is faster than extracellular movement, then the result could be faster rates of phage movement-driven plaque growth than may be achieved with phage motion instead consisting mostly of extracellular diffusion.

Not all adsorptions will result in such intracellular movement toward a plaque’s periphery since not all bacteria will be oriented as a pathway away from a plaque’s center. On the other hand, initial adsorptions of bacteria will tend to be closer to a plaque’s center than its periphery due simply to the fact that plaques grow as phages diffuse away from their centers [76], i.e., such that phage intracellular movement up ‘ladders’ may be more likely than phage movement down ‘chutes’. This proposed bias, while it might further increase rates of plaque development due to outward intracellular phage movement being more likely than inward movement, should nonetheless not represent a requirement for the described ‘Chutes and Ladders’ effect. 

**Figure 5:**
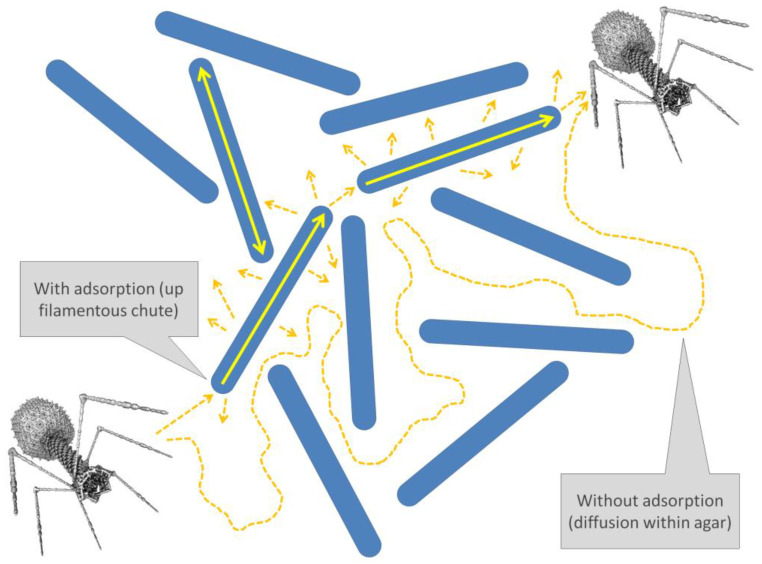
Proposed phage use of filamentous bacteria as a means of speeding up plaque growth. If bacteria are long enough, latent periods short enough, and diffusion (dashed orange arrows) through agar slow enough, then infection of appropriately oriented bacteria could serve as ‘ladders’ (solid yellow arrows) for movement of phages faster or deeper into a bacterial lawn than virions may be able to progress extracellularly by diffusion alone.

Although relatively easy to at least visualize within a context of phage plaque formation (Figure 5), it is difficult to say to what extent similar biochemical phage movement within bacteria might occur about and below the surfaces of biofilms. If such movement was possible, with filamentous bacteria for example able to enhance phage penetration deeper into clonal biofilms containing those bacteria, then this could serve as another evolutionary disincentive for bacteria within biofilms to have cell lengths that are any greater than otherwise might be needed. This would be besides disincentives resulting from such bacteria serving simply as larger targets for phage adsorption.

### 4.6. Phage–Antibiotic Synergy

One interesting consequence of this proposed chutes-and-ladders mechanism could be contribution to a suite of phenomena known collectively as phage–antibiotic synergy (PAS) [78,79,80,81,82]. In PAS, phages can display greater infection activities especially in the presence of sub-inhibitory concentrations of antibiotics. These are insufficient antibiotic quantities to result in blocking of the growth of bacterial populations, with one measure of improved phage-infection activity being larger plaque sizes, e.g., [83,84,85,86,87]. Sub-inhibitory concentrations of certain antibiotics are also known to give rise to bacterial filamentation [88,89], though not in all bacteria [90] nor to equivalent extents with different antibiotics [91]. Filamentation, however, is likely not the only correlate to increased plaque sizes associated with bacterial lawn exposure to sub-inhibitory concentrations of antibiotic [85,91]. In addition, it is important to keep in mind when interpreting PAS experiments, e.g., [84], that often antibiotic inhibitory concentrations (i.e., minimum inhibitory concentrations, MICs) are determined under different conditions [92] from those employed during plaque formation. Notwithstanding these various discrepancies, it is bacterial filamentation given exposure to sub-inhibitory concentrations of antibiotic, as well as mechanisms that can increase the amount to time phages spend diffusing, which are the focus of this section. 

In the original PAS study [83], in addition to bigger plaque sizes, larger burst sizes (phages produced per phage-infected bacterium) were seen as well in the presence of antibiotic than without. Phages have also been shown to display shorter latent periods (per-cell duration of phage infections) given bacterial filamentation [93], in both of these cases determined as based on one-step growth experiments [11,94]. The larger burst sizes are perhaps not surprising [95] given the larger sizes of filamentous bacteria; and accelerated lysis as an aspect of PAS has also been reported elsewhere [81]. Alternatively, these larger burst sizes can be correlated instead with modestly longer phage latent periods [85]. Larger burst sizes as a component of PAS was reported also in [96], and larger phage burst sizes in particular are thought to be able to result in larger phage plaques [64].

Another possible explanation for larger plaque sizes with antibiotic exposure is that the potential for phages to display lysis inhibition may be reduced given infection of filamentous bacteria [93]. Lysis inhibition is a phenomenon of inducibly extended phage latent periods that gives rise to at least a visual perception that plaques are smaller relative to when lysis inhibition is absent [15]. Lysis inhibition nonetheless likely is relatively rare among phages. On the other hand, antibiotic interference with display of lysis inhibition might help to explain the greater plaque sizes observed by Ryan et al. [97] working with coliphage T4 as well as some of the enlarged plaques seen by Comeau et al. [83] (coliphages T4, RB32, and RB33). It is possible as well that by slowing bacterial replication, sub-inhibitory concentrations of antibiotics could contribute to increased plaque size simply by giving phages more time to diffuse outward and infect bacteria prior to entrance of the bacterial lawn into stationary phase [86], as also may be accomplished by UV irradiating indicator bacteria prior to initiating a lawn [98]. That is, with larger phage plaques resulting from plaque growth occurring over longer time periods rather than larger plaques necessarily being due to faster plaque growth, though both phenomena (faster and longer growth) each can give rise to an observation of larger plaques. Alternatively, slowing bacterial lawn growth should also expose phages to fewer bacteria longer, resulting in less sorptive scavenging (Section 4.4) and thereby potentially more time diffusing rather than infecting, again giving rise to larger plaques.

Bacterial infection of filamentous bacteria, as an aspect of PAS, might also allow for more rapid phage movement toward the periphery of plaques, i.e., as due to the above-hypothesized chutes-and-ladders mechanism. Yet other mechanisms have also been proposed to explain the PAS phenomenon, particularly ones that consider how phage presence can make bacteria more susceptible to antibiotics [80]. Chutes and ladders, as a means of bypassing limitations to virion diffusion within agar, nonetheless at least potentially could contribute to increased plaque growth rates in association with bacterial filamentation. That PAS aspect—as well as shorter phage latent periods, larger phage burst sizes, and slower lawn growth—together could result in more time or more phages moving toward the periphery of plaques when in the presence of sub-inhibitory concentrations of antibiotics, rather their spending more time infecting smaller, motionless bacteria.

## 5. Adsorption Rate Heterogeneity

Additional phenomena can affect rates at which phages can adsorb bacteria. In this section, we consider various forms of heterogeneity, whether they are phenotypic heterogeneity across populations (without necessarily also environmental heterogeneity), heterogeneity in population densities across environments, or additional aspects of environmental heterogeneities. I consider each separately for phages and bacteria as well as for the different categories, though there is little reason that any one type of heterogeneity might exclude another. The general argument is that variation can exist in terms of rates that individual phages adsorb as well as individual bacteria are adsorbed, variations that are independent of rates of movement as considered instead in Section 3 and Section 4. See Table 1 for a summary of which of the following sections cover what.

### 5.1. Bacterial Heterogeneity across Populations

Bacterial phenotypic heterogeneity can exist even given seeming genetic and environmental homogeneity [99]. From a perspective of phage adsorption rates, especially relevant can be variations in cell size (*R*) or in cell adsorbability given virion encounter (*f*). Our expectation in particular is that bacteria that are about to divide will be more likely to encounter a phage than a bacterium that has just divided, given their roughly two-fold difference in size. Though not strictly in terms of the size of cells, bacteria that have formed into microcolonies can also vary across bacterial populations in terms of size, with larger microcolonies, as made up of a greater number of cells, e.g., such as due to earlier microcolony initiation, presumably more likely to encounter phages than smaller microcolonies [100]. What can come next with these microcolonies after phage encounter is considered in Section 5.5 and Section 5.6.

Alternatively, Ge et al. [21] provide an overview of “Strategies of preventing phage adsorption by host” (p. 4). Particularly relevant here could be the production of glycocalyx, small-molecule inhibitors of adsorption receptor molecules, and adsorption decoys such outer membrane vesicles. Generally speaking, if these strategies were to impact individual bacteria unevenly, then that could also lead to bacterial adsorption heterogeneity. Another possibility is variation in phage receptor densities on bacterial surfaces, with display of lower densities thought to result in lower likelihoods of transition from phage encounter to phage irreversible attachment [30,37]. In addition, there can exist differences among bacteria that are found within a single population in terms of whether a potential phage receptor is present at all. For example, this could be in terms of phase variation [101,102,103] or only intermittent bacteria display of phage-adsorbing conjugation pili [104,105].

Overall, there could be enrichment for smaller or otherwise less readily adsorbed cells within bacterial populations due to those cells displaying a greater potential to avoid becoming phage adsorbed. To a degree, however, we should have an expectation that heterogeneity in terms of adsorbability, at least so long as phage receptors are still present on all bacteria to some degree, can be overwhelmed given the exposure of bacteria to greater phage titers, e.g., such as 10^8^ phages or more per mL during phage therapies [7] (Appendix A.4.2).

### 5.2. Bacterial Heterogeneity as a Function of Environments

Bacterial heterogeneity as a function of environmental conditions can occur either across space or across time. Conditions that result in slower bacterial growth or bacteria entrance into stationary phase, for example, can vary from location to location or over the course of the equivalent of a standard bacterial growth curve. Contrasting the physiological heterogeneity considered in the previous section, however, constant environmental conditions in this scenario are not assumed. In any case, the consequence of slower or no bacterial growth can be smaller cells and thereby slower or less adsorption, a phenomenon that has been described by various authors [9,106,107,108,109]. 

We can also consider variation in bacterial physiologies that occur as a function of bacterial clustering into microcolonies and biofilms. One pertinent example is variation in the extent of bacterial display of receptors as a function of environmental circumstances such as reductions associated with quorum sensing [110]. I argue elsewhere, however, that under conditions facilitating quorum sensing, as well as resulting in reduced rates of bacterial growth within biofilms, bacterial densities may be sufficiently high that decreased phage adsorption rate constants may not have a substantial impact on the likelihood that a given phage will succeed in adsorbing [38] (see, similarly, as summarized in Figure 4).

### 5.3. Phage Heterogeneity across Populations

**Figure 6:**
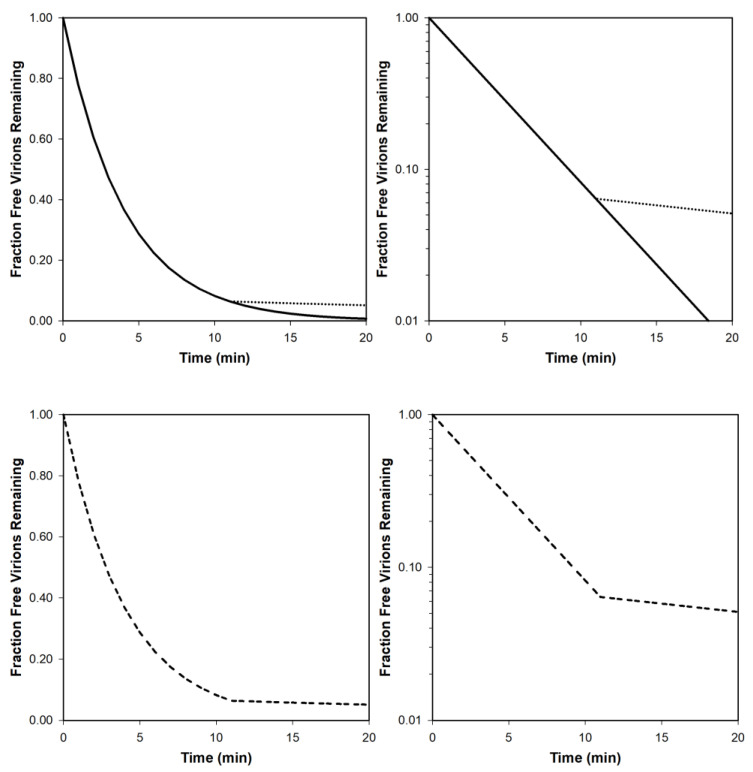
Visualization of theoretical phage adsorption curves, including biphasic curves. The two top curves differ only in terms of the scale of their *y*-axes, which is linear to the left and logarithmic to the right. The adsorption rate constant, *k*, has been set to 2.5 × 10^−9^ mL^−1^ min^−1^ [8], for the solid-line curves, while *N* has been set to 10^8^ cells per mL. For the dotted-line curves, k instead has been arbitrarily set to 2.5 × 10^−10^ mL^−1^ min^−1^, with these latter curves initiated at their point of intersection with the solid-line curves. If these were biphasic curves, then they would be represented as seen on the bottom, shown there as dashed lines, which are identical to the top curves except that the *k* = 2.5 × 10^−9^ mL^−1^ min^−1^ curves are not shown below the point of intersection of the two curves on top. Note in particular how with the curve shown in the lower-left it may be difficult to appreciate that biphasic adsorption is occurring whereas with the otherwise identical curve shown to the lower-right the biphasic nature of curves can be more obvious. These graphs were generated as [Faction Free Virions Remaining] = e^−*kNt*^, where *N*, as bacterial concentration, is equivalent to *N*_0_ in Equation (A6).

Our expectation given homogenous phage and bacterial populations is for free phages to display exponential declines in population sizes over time as they adsorb bacteria. The result should be a straight line as graphed log-linearly [11] (Figure 6, upper-right). If graphed without a log-transformed *y* access, such curves instead have long tails, asymptotically heading toward zero phages (Figure 6, upper-left). The latter in and of itself therefore should not be viewed as deviating from constant rates of phage adsorption. 

Alternatively, it is possible for such curves when graphed log-linearly to be biphasic, with an initially more rapid rate of decline in numbers of unadsorbed phages (or rate of increase in adsorbed phages) that is followed by a slower rate of loss of unadsorbed phages (Figure 6, lower curves). The latter can be a consequence of a portion of the phage population consisting of virions that simply are phenotypically inherently slower adsorbers, presumably due to temporary differences in the affinity those phages have for bacterial surfaces [25]. For instance, there could exist differences in the extent to which phage tail fibers are in an adsorption-ready state, an issue considered more in the following section, e.g., as may be observed particularly given borderline sub-optimal adsorption conditions [57]. 

Similarly though more extreme, Storms et al. [23,111] describe an ‘adsorption efficiency’, which is the fraction of a phage population during an adsorption experiment which irreversibly attach at all. Delbrück [9] called those other phages that fail to attach, ‘residual free phage’, while Storms and Sauvageau [112] review the use of an equivalent ‘residual fraction’ (see too [113]), which possess “a physiological defect hindering their adsorption capabilities” ([25], p. 358). Thus, a heterogeneity can exist within phage populations during assays of adsorption rates, with a faster adsorbing phage population coexisting with either a slow or not adsorbing phage population. Furthermore, it is possible that in at least some cases this heterogeneity is genotypic rather than just phenotypic [112].

### 5.4. Phage Heterogeneity as a Function of Environments

It is well known that phages can require different adsorption cofactors to successfully attach to otherwise adsorption-susceptible bacteria, e.g., [58,111]. These adsorption cofactors commonly include divalent cations such as calcium ions but also monovalent cations such as those of sodium or potassium. So too there exist organic adsorption cofactors, particularly as has been observed for tryptophan [33,57,111], which may allow certain coliphages to distinguish between colonic and extra-colonic environments [32,114]. See especially Conley and Wood [58] for a description of the utility that these and other factors, such as temperature, could have toward allowing coliphage T4 to vary its adsorption ability and thereby adsorption rates (see too [114]). 

For these latter phages, the distinction between being more adsorption competent and less adsorption competent has to do with the conformation of their long tail fibers, which when fully extended (not retracted) are ready to interact with cell surfaces with their tips whereas alternatively they can be bound back (retracted) in a manner that makes such interactions with bacterial surfaces less likely [57]. As noted, it is conceivable that heterogeneity within phage populations in terms of virion adsorption rates (previous section) could be a consequence of degrees of extension of virion tail fibers prior to encounter with otherwise adsorption-susceptible bacteria.

### 5.5. Heterogeneity in Bacterial Concentrations

The clustering of bacteria into microcolonies and biofilms has two consequences that can be relevant to rates of phage adsorption. One has to do with variations in the concentrations of bacteria across environments, while the other has to do with variations in the potential for bacteria to be impacted by quorum sensing [115] or other means by which bacterial physiology may be modified if packed closely together (e.g., for the latter, as due to reduced access to nutrients and oxygen [116]). Quorum sensing as noted can be considered also as a driver of bacterial heterogeneity across environments (Section 5.2), while clustering similarly can lead to bacteria that are found more toward the interior of microcolonies and biofilms having more stationary phase-like physiologies [117] (see [74] for review of the possible impact of these changes in physiologies on phages). In this section, though, the focus is on variation simply in concentrations of bacteria across environments.

The consequence of variation in bacterial concentrations across environments means that the likelihood that a phage will encounter a bacterium will vary as a function of where that phage is located. Thus, virions that are further from clusters of bacteria—resulting thereby in lower local densities of potential host bacteria—will tend to take longer to encounter a susceptible bacterium than phages that are located closer to clusters. These longer extracellular search durations can potentially result in greater likelihoods of virion inactivation prior to adsorption occurring [1,118]. So too, phages can diffuse away from clusters, thereby increasing the length of time until subsequent bacterial encounter by these phages is likely to take [42]. Alternatively, phages which are found close to clusters of bacteria should succeed in adsorbing over shorter time periods.

The latter means that phages which are released within the vicinity of such clusters will, at least prior to their diffusing or flowing away, have a higher likelihood of encountering a susceptible bacterium than a phage that is released into a spatially homogeneous environment (holding total bacterial numbers constant). This has the effect of making clusters of bacteria potentially more vulnerable to exploitation by phages, once an initial adsorption has occurred, than individual, non-clustered bacteria. Previously I have dubbed this a “Spatial vulnerability” [51], and this greater vulnerability should be the case unless such clusters possess better defenses against phages than non-clustered bacteria [74]. Also resulting in greater vulnerability should be the noted larger size of such clusters relative to individual cells, making it more likely that some phage will encounter at least one of the constituent cells [100].

Note that concerns over whether phages, due to their locations, can take more time before they adsorb while other phages take less time may be ecologically relevant for individual phages, but should be of less relevance during phage therapies. This is at least so long as targeted bacteria are all similarly reachable by dosed phages, and these phages are supplied in reasonably large numbers (e.g., such 10^7^/mL and higher), so that at least some of the supplied virions are reasonably likely to encounter susceptible bacteria regardless of variations in bacterial concentrations. Alternatively, especially if phages cannot be easily applied directly to targeted bacteria, then issues of phages taking longer to reach those bacteria, because virions are starting out their searches from locations that are quite far away, can be an issue. This is particularly so to the extent that barriers to virion movement exist, e.g., such as obstacles to phage absorption into the blood or phage distribution to body tissues starting from the blood [119]. Fortunately, however, this concern to a large extent may be reduced as an absolute impediment to successful phage treatment due to the potential for phages to exist across environments also with heterogeneous concentrations.

### 5.6. Heterogeneity in Phage Concentrations

The ability of phages to amplify their numbers in situ represents one of their key attributes as therapeutics, especially toward combating bacterial infections to which phages cannot easily be directly applied. The result of highly localized phage amplification in particular can be a substantial heterogeneity in phage concentrations across environments such as within phage-treated bodies. Resulting high or at least relatively high phage titers should allow for more effective exploitation of localized clusters of especially clonally related bacteria, that is, with locally higher phage titers allowing for more rapid adsorption of those bacteria. 

More generally, the concept of “Active phage therapy” has been used to describe phage treatments that are reliant on phage in situ replication [120,121,122] to achieve inundative phage concentrations [7]. I have suggested, however, that such active treatment should be distinguished into more global vs. more localized aspects [100], with localized active treatment dependent especially on slow movement of newly produced virions away from regions of bacterial clustering. One way to view this potentially greater localized phage impact, though from a more ecological rather than applied (phage therapy) perspective, is in terms of what I describe as foci of phage infection [42]. These can be regarded as the biofilm-associated equivalent of phage plaque growth [42]. Both foci of infection and localized phage population growth within plaques occur as a consequence of only limited movement of phages away from their point of release from infected bacteria, or at least not instantaneous movement away. This is in combination with high densities of phage-exploitable bacteria being found in the immediate vicinity of those newly released phages, i.e., as making up single-species biofilms, clonal microcolonies, or bacterial lawns. Bacteria thus can be eliminated only locally in the course of, e.g., plaque development due to phages replicating to higher titers again only locally. The contrast would be consequences of phage replication within environments consisting instead of well-mixed broth media.

A further consequence of localized phage replication can be their “Active penetration” into biofilms [122]. This mechanism probably is particularly dependent on phage-mediated lysis of targeted bacteria. The result should be phages being able to move from more biofilm surface-associated bacterial targets to bacteria that are found below these surfaces. That is, those below-surface bacteria may initially be protected from phages by sorptive scavenging effected by still-intact overlying bacteria (Section 4.4), but given active penetration that protection would be only temporary.

## 6. Conclusions

The faster that phages are able to reach bacteria, then the sooner that phages can have an impact on those bacteria. At its most basic, we can predict to what extent that rapidity might occur based upon a combination of various properties, collectively describing a phage’s adsorption rate constant (Section 2), though relevant as well are phage and bacterial concentrations (Figure 4 and Appendix A). Generally speaking, the greater the concentration of bacteria, then the sooner that free phages will be lost to adsorption whereas the greater the concentration of phages, then the faster bacteria will become phage adsorbed.

Though this basic process of phage acquisition of bacteria via adsorption is driven by virion diffusion, other forms of movement can also impact the rate at which adsorption can take place, particularly when the motions of phages and bacteria are relative to each other (Section 3). So, too, however, various factors can affect simply the rate of phage movement via diffusion (Section 4). Environments, concentrations, and phage or bacterial properties also can vary across either time or space in ways that can impact rates that phages adsorb bacteria (Section 5).

Notwithstanding these numerous factors, often the most easily manipulated means of modifying rates that bacteria are reached by phages is to either supply more phages in phage therapy treatments or instead to use phages that have a greater potential of increasing their numbers in situ in the course of infecting targeted bacteria. The latter, though, requires greater knowledge of potential phage–bacterial interactions, particularly as they may occur in situ, than may be readily obtained in the course of routine phage treatments. Therefore, to assure that phages succeed in reaching bacteria with desirable but not necessarily excessive kinetics, the simplest approach may be to start with relatively low phage titers, e.g., such as 10^7^ per mL achieved in situ, and then raise those numbers as required, such as increasing titers to 10^8^ per mL via dosing and so on over the course of days until the desired degrees of phage adsorption, and thereby bacterial infection and killing, are achieved.

## Figures and Tables

**Table 1:** Definitions of terms relevant to phage adsorption rates.

Term *	Definition
Adsorption process	Multi-step progression that involves a combination of virion diffusion, phage encounter with a bacterium, and then various processes of attachment to that bacterium
Adsorption rate (*dP*/*dt*)	Description of the timing particularly of irreversible virion attachment to bacterial cells, as is a function of the phage adsorption rate constant, phage concentrations, and bacterial concentrations
Adsorption rate constant (*k*)	Description of the intrinsic timing of virion attachment, particularly irreversible attachment to bacteria
Attachment	Post-encounter, specific interactions of a virion with a bacterial surface
Bacterial concentration (*N*)	Description of numbers of bacteria within an environment such as in per mL units
Collision kernel	Description of the number of collisions expected between particles within a given volume over a given span of time, as involves a particle’s rate of motion, its size, and between-particle affinity
Diffusion rate (2) (*C*)	Random virion motion within and relative to fluid environments; this is a key aspect of particle motion in predicting adsorption rate constants
Efficiency (of attachment) (*f*)	Description of the affinity between particles, such as between a phage virion and targeted bacterium, with affinities ranging from 0 to 1; this otherwise can be described as a collision efficiency in predicting adsorption rate constants
Encounter (Collision) (3)	Contact of an extracellular virion with an object such as an adsorbable bacterium
Extracellular search (2)	Period starting with phage virion release from a phage-infected bacterium and potentially ending with virion encounter with an adsorbable bacterium
Free phage (or free virion)	Extracellular phage particle, contrasting with virions existing prior to their release; the adsorption process involves the conversion of free phages to irreversibly attached virions
Genome translocation (6)	Movement of, until-this-point, virion-encapsidated phage chromosome across the bacterial cell envelope, from the extracellular virion particle into the bacterial cytoplasm
Infection (0)	Here defined as a state involving, minimally, the presence of a phage genome within a bacterium’s cytoplasm; the process of “Infection” should not be equated with the process of “Adsorption”, e.g., given the existence of superinfection exclusion
Irreversible attachment (5)	Committed interactions between a virion and bacterial surface as ideally (for the phage) leading to genome translocation
Phage receptor	Bacterial molecule, such as a protein or polysaccharide, that is displayed on the outside of a bacterium’s cell envelopment and to which a virion displays affinity in the course of attachment (reversible or irreversible)
Release (1)	Transition of an intracellularly located virion particle into an extracellularly located virion; the process of becoming a free phage or free virion
Reversible attachment (4)	Non-covalent initial interactions between a virion and a bacterial surface; generally followed by either irreversible virion attachment or instead by virion desorption
Superinfection exclusion	Process of blockage of phage genome translocation that acts following virion irreversible attachment
Target radius (*R*)	As controls in part the likelihood of encounter (or collision) between particles, with a larger radius resulting in greater likelihoods of collision; it generally is only the bacterium’s radius rather than that of virions as well which is considered
Titer (*P*)	Description of the concentration of free phages within an environment, such as in per mL units

* Parenthetical numbers are equivalent to as indicated in the main text (Figure 2). Parenthetical italicized letters are variable or parameter name abbreviations, except for the symbol, *d*, which stands for ordinary derivative. Note that some of the presented symbols are used here exclusively in Appendix A.

**Figure A1:**
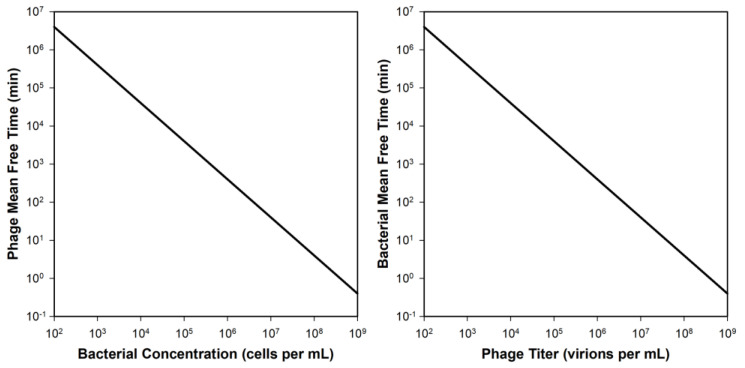
Mean free times (average time until adsorption) as a function of bacterial (**left**) or phage (**right**) concentrations (*N* and *P*, respectively). The adsorption rate constant (*k*) is defined here as equal to 2.5 × 10^−9^ mL^−1^ min^−1^ from Stent [8] and mean free times are defined as 1/*kN* [124], i.e., “on average, the time spent finding a host”, p. 547 [125], or 1/*kP*, respectively. These numbers also approximate the time it takes for half of a population (of free phages or unadsorbed bacteria) to be potentially lost to adsorption, thus loosely representing half-lives [7]. See too the equivalent Equation (15) of [24].

## Data Availability

Data sharing not applicable.

## Appendix A. Dependence of Adsorption Rates on Phage and Bacterial Concentrations

In this appendix, I consider theoretically the claims made in Figure 4, involving the impacts of differences in phage or bacterial concentrations on phage adsorption rates.

### Appendix A.1. Phage Adsorption Rate Theory

To derive a theory of phage adsorption rates, we can start with this differential equation [123]:

(A1) $$\frac{dP}{dt}=-kNP.$$

In words, this indicates that the instantaneous change in phage concentration (*P*) as a function of time (*t*) is equal to the opposite of the product, in order, of the adsorption rate constant (*k*), the bacterial concentration (*N*), and again the phage concentration (titer). Thus, the greater the negative value on the right, then the faster the instantaneous rate of decrease in absolute phage numbers as described on the left; that is, the greater the total number of phage adsorptions per unit time. The variable abbreviations are as employed by Stent [8].

Note from the above equation that instantaneous rates of overall phage adsorption will increase as a linear function of both phage and bacterial concentrations as well as by a linear function of the magnitude of the phage adsorption rate constant. Those issues are crucial to understanding phage biology, phage ecology, phage therapy, and phage-mediated biological control of bacteria since they help to determine, in combination with phage latent periods and burst sizes [11], the rate at which a phage population increases in number, i.e., its growth. Thus, phages will adsorb faster when bacterial concentrations are higher. Indeed, with each ten-fold increase in bacterial concentration, then a given phage will adsorb at a rate that is ten-fold faster. This is equivalent to stating that a population‘s growth will increase as a function of concentrations of a limiting resource, in this case with this limiting resource being bacteria to infect. Note, though, that the measurement here is one of how long a phage remains free, that is, not adsorbed, and thus ten-fold faster means that the duration a virion remains free is ten-fold shorter, e.g., 1000 s vs. 100 s vs. 10 s vs. 1 s, and so on (Figure A1, left panel). See, though, Stent and Wollman [31] for considerations of how real-world adsorption rates can be complicated at very high bacterial densities, such as above 10^8^/mL.

Similarly, there will be more phage adsorptions per unit time when more phages are present, with a ten-fold increase in phage numbers resulting in a ten-fold increase in the rate of occurrence of phage adsorptions (as implied in Figure A1, right panel). The latter is part of the basis of phage populations, in the absence of limits, tending to increase in number exponentially over time: the more phages that are present, then the more phage infections that will occur. Producing more free phages therefore results in more phage infections. Less intuitively, however, the rate at which a given phage adsorbs is less important to phage therapy success than the rate at which a given bacterium becomes phage adsorbed. This distinction between what controls rates that a phage adsorbs vs. rates that a bacterium becomes adsorbed is addressed in the following two sections.

### Appendix A.2. Rate of Loss of Individual Virions to Adsorption

The phage concentration in Equation (A1), as indicated by *P* on the right side, is there because the left side of the equation refers to the overall rate of phage population adsorption. This is rather than referring to the rate of adsorption by individual phages. That distinction is fairly subtle—between population rates of phage adsorption and individual-phage rates of adsorption—but nonetheless is both real and relevant, as this section considers.

As noted, the population rate of phage adsorption will be greater, in a linear fashion, the greater the current phage concentration, i.e., *P*, or instead *P*_0_ as presented further below. The rate of adsorption by individual phages, however, will not appreciably vary as a function of overall phage concentrations, at least so long as the bacterial population is still well below its overall numerical capacity to adsorb phages [12,106]. The latter is something that can be described as a bacterium’s ‘saturation capacity’ [123] or ‘adsorption capacity’ [8], and see also [30].

To explore the rate of adsorption of single phage particles, rather than the overall rate of phage adsorptions within a given environment, then Equation (A1) instead would be presented as,

(A2) $$\frac{dP}{dt}=-kN1,$$

i.e., replacing *P* with 1, for one phage. Or, technically, one phage per whatever volume units *k* is defined using, such as per one mL (indeed, for the sake of the following argument, one can replace *P* with whatever number one likes, just so long as it is a constant rather than a variable). The rate at which a given individual phage is expected to adsorb thus is expected to change as a function of the phage adsorption rate constant and the concentration of bacteria [1], but not as a function of phage titers. In other words, intuitively, the more bacteria that are present to adsorb (*N*), then the sooner that individual phages will find bacteria to adsorb, i.e., as summarized in Figure 4.

### Appendix A.3. Rate of Loss of Individual Bacteria to Phage Adsorption

Contrasting individual phages, the rate at which individual bacteria are found by phages is indeed a function of phage titers. That is,

(A3) $$\frac{dU_{N}}{dt}=-k1P,$$

where *U_N_* refers to the concentration of uninfected (i.e., *U*nadsorbed) bacteria, e.g., as may be measured in terms of CFUs (colony-forming units) per mL.

Intuitively, this equation indicates that when more phages (as virions) are present, then bacteria will become phage adsorbed faster (as also summarized in Figure 4). Thus, if ten-fold more phages are present within an environment, then uninfected bacteria should be lost to phage adsorptions ten-fold faster. The corollary is that if phage titers are, say, 1000-fold lower, e.g., 10^5^/mL vs. 10^8^/mL, then phage-uninfected bacteria will become phage-infected bacteria at a rate that is 1000-fold slower.

Note in Equations (A2) and (A3) that what is being described are instantaneous rates, particularly without taking into account declines in either phage or bacterial numbers over time. Declines in numbers of phage virions, as these adsorb bacteria, are relevant to Equation (A3) because as *P* becomes smaller, then so too does the instantaneous rate of phage adsorption to these bacteria. An equivalent phenomenon would occur with Equation (A2) for reductions in *N* over time, though typically adsorbable bacteria do not appreciably decline in number at least instantaneously upon adsorption. This is due to the typical ability of bacteria to adsorb more than one phage at a time.

Of importance to phage therapy, we can restate the latter point as indicating that not all phage adsorptions occur to bacteria that are not-yet phage infected. In particular, these phage adsorptions to already infected bacteria, though with some exceptions due to changes in infection physiologies [15], are not expected to contribute to reductions in bacterial numbers over time.

### Appendix A.4. Once More, but without Calculus

The above differential equations are easily solved, which for Equation (A1) is as follows [8,123],

(A4) $$P=e^{-kN_{0}t}P_{0},$$

where *P*_0_ is the phage concentration at the start of some interval, of length, *t*, and *N*_0_ is simply the starting concentration of targeted bacteria, particularly as seen at the same zero point. *P* on the left side of the equation may more legitimately be labeled as *P_t_*, meaning *P* at time, *t*. Thus, we have,

(A5) $$P_{t}=e^{-kN_{0}t}P_{0}.$$

#### Appendix A.4.1. Fraction of Phages Adsorbing

Equation (A5) is not a description of instantaneous rates of phage adsorption but instead is a description of the total number of free phages that are expected to remain after some period of adsorption, *t*, given a starting titer of *P*_0_ and holding bacterial concentrations constant over that time. More generally, the fraction of phages remaining *U*nadsorbed after this period of adsorption, *U_P_*, would be defined as,

(A6) $$U_{P}=\frac{P_{t}}{P_{0}}=e^{-kN_{0}t}.$$

The total fraction of phages that have *A*dsorbed (*A_P_*) thus is 1 minus *U_P_*, or

(A7) $$A_{P}=1-\frac{P_{t}}{P_{0}}={1-e}^{-kN_{0}t}.$$

Therefore, the greater the phage adsorption constant (*k*), the more bacteria that are present (*N*_0_), and the more time (*t*), or indeed, the more phages that are present from the start of the adsorption period (*P*_0_), then the greater the *T*otal number of phages per mL that will be expected to have adsorbed. This is described as follows:

(A8) $$T_{P}=P_{0}A_{P}=P_{0}\left({1-e}^{-kN_{0}t}\right).$$

#### Appendix A.4.2. Fraction of Bacteria Adsorbed

The fraction of phages that remain unadsorbed is not identical to the fraction of bacteria that remain unadsorbed. Furthermore, the fraction of bacteria remaining unadsorbed is more directly relevant to phage therapy success than the fraction of added phages that either do or do not adsorb. Analogous to Equation (A4), the fraction of bacteria remaining unadsorbed is described as,

(A9) $$N_{t}=e^{-kP_{0}t}N_{0},$$

where *N*_0_ again is the starting bacterial concentration and *N_t_* is the concentration of unadsorbed bacteria remaining after being incubated for a duration of time, *t*, in the presence of a titer of *P*_0_ phages.

Similar to as above for phages (previous section), we can then define the fraction of bacteria which remain unadsorbed (*U_N_*), the fraction of bacteria which are adsorbed (*A_N_*), and the total number of bacteria we will expect to have been adsorbed (*T_N_*):

(A10) $$U_{N}=\frac{N_{t}}{N_{0}}=e^{-kP_{0}t},$$

(A11) $$A_{N}=1-\frac{N_{t}}{N_{0}}={1-e}^{-kP_{0}t},$$

(A12) $$T_{N}={N_{0}A_{N}=N}_{0}\left({1-e}^{-kP_{0}t}\right),$$

The rate at which a given population of bacteria of *N*_0_ concentration is found by phages thus is a function of a combination of the phage adsorption rate constant, time, and the phage titer, the latter particularly as found at the start of adsorption periods (*P*_0_).

The percentage of bacteria that have not been phage adsorbed, assuming that phage titers are held constant over time (Equation A10), can be calculated for various adsorption intervals. For instance, with *k* defined as 2.5 × 10^−9^ mL^−1^ min^−1^ per Stent [8] for coliphage T4, given a 30-min adsorption period and only 10^7^ phages/mL, then nearly half of the bacteria will remain unadsorbed. For a 10-min adsorption period, however, 92% of bacteria have become phage adsorbed starting with a phage titer of 10^8^/mL (i.e., 8% remaining unadsorbed). The adsorption rate constant, *k*, for other phages, though, can be considerably higher. For example, it is approximately 10^−8^ mL^−1^ min^−1^ for phage λ [126]. This would be expected to give rise to faster reductions in numbers of unadsorbed bacteria, i.e., four-fold greater than as calculated for phage T4 in the previous paragraph. It is also possible for *k* instead to be considerably smaller, e.g., such as 10^−10^ mL^−1^ min^−1^ or even 10^−11^ mL^−1^ min^−1^ [127,128,129,130], thereby resulting in substantially slower reductions in unadsorbed bacteria than as indicated above. To explore the impact of such differences in adsorption rate constants on bacterial survival over time, in Table A1, Table A2, Table A3 and Table A4 that survival has been calculated for *k* set equal to 10^−8^, 10^−9^, 10^−10^, and 10^−11^ mL^−1^ min^−1^, respectively.

Alternatively, especially with higher bacterial densities, there can be an expectation that titers of free phages would decline somewhat over time due to adsorption to bacteria. The result of such declines in *P* with time would be less of a phage impact on bacteria than as indicated in those tables, unless free phage losses to adsorption (i) are balanced by phage replication and associated release of new free phages, (ii) are minimized by exposure to large, well-mixed volumes of phages (thereby reducing total phage losses to adsorption over time), or (iii) instead are countered by repeated or continuous phage dosing. Keep in mind too that in situ rates of phage adsorption may not be identical to in vitro-determined rates.

**Table A1:** Percentage of bacteria remaining unadsorbed as functions of phage titers (*P*) and adsorption intervals (*t*), *k* = 1 × 10^−8^ mL^−1^ min^−1^ *.

*t* →	1	5	10	30	60	120	180	240	300
*P* ↓	Unadsorbed bacteria (percentage) as based on Equation (A10):								
10^5^	100%	100%	99%	97%	94%	89%	84%	79%	74%
10^6^	99%	95%	**90%**	74%	55%	30%	17%	9%	5%
10^7^	**90%**	61%	37%	5%	0%	0%	0%	0%	0%
10^8^	37%	1%	0%	0%	0%	0%	0%	0%	0%
10^9^	0%	0%	0%	0%	0%	0%	0%	0%	0%
10^10^	0%	0%	0%	0%	0%	0%	0%	0%	0%

* Time (*t*) is in minutes and phage titers (*P*) are in per mL units. Percentage of unadsorbed bacteria (*U_N_*) are calculated as equal to e^−*kPt*^ and have been rounded to the nearest integer. Here, 90% reductions have been emphasized to aid in comparisons between tables.

**Table A2:** Percentage of bacteria remaining unadsorbed as functions of phage titers (*P*) and adsorption intervals (*t*), *k* = 1 × 10^−9^ mL^−1^ min^−1^.

*t* →	1	5	10	30	60	120	180	240	300
*P* ↓	Unadsorbed bacteria (percentage) as based on Equation (A10):								
10^5^	100%	100%	100%	100%	99%	99%	98%	98%	97%
10^6^	100%	100%	99%	97%	94%	89%	84%	79%	74%
10^7^	99%	95%	**90%**	74%	55%	30%	17%	9%	5%
10^8^	**90%**	61%	37%	5%	0%	0%	0%	0%	0%
10^9^	37%	1%	0%	0%	0%	0%	0%	0%	0%
10^10^	0%	0%	0%	0%	0%	0%	0%	0%	0%

**Table A3:** Percentage of bacteria remaining unadsorbed as functions of phage titers (*P*) and adsorption intervals (*t*), *k* = 2.5 × 10^−10^ mL^−1^ min^−1^.

*t* →	1	5	10	30	60	120	180	240	300
*P* ↓	Unadsorbed bacteria (percentage) as based on Equation (A10):								
10^5^	100%	100%	100%	100%	100%	100%	100%	100%	100%
10^6^	100%	100%	100%	100%	99%	99%	98%	98%	97%
10^7^	100%	100%	99%	97%	94%	89%	84%	79%	74%
10^8^	99%	95%	**90%**	74%	55%	30%	17%	9%	5%
10^9^	**90%**	61%	37%	5%	0%	0%	0%	0%	0%
10^10^	37%	1%	0%	0%	0%	0%	0%	0%	0%

**Table A4:** Percentage of bacteria remaining unadsorbed as functions of phage titers (*P*) and adsorption intervals (*t*), *k* = 2.5 × 10^−11^ mL^−1^ min^−1^.

*t* →	1	5	10	30	60	120	180	240	300
*P* ↓	Unadsorbed bacteria (percentage) as based on Equation (A10):								
10^5^	100%	100%	100%	100%	100%	100%	100%	100%	100%
10^6^	100%	100%	100%	100%	100%	100%	100%	100%	100%
10^7^	100%	100%	100%	100%	99%	99%	98%	98%	97%
10^8^	100%	100%	99%	97%	94%	89%	84%	79%	74%
10^9^	99%	95%	**90%**	74%	55%	30%	17%	9%	5%
10^10^	**90%**	61%	37%	5%	0%	0%	0%	0%	0%

#### Appendix A.4.3. Not All Phage Adsorptions Are to Not Yet Adsorbed Bacteria

As noted (Appendix A.2), not all phage adsorptions will be to bacteria that are not yet phage adsorbed. Thus, we have an expectation that *T_P_* will always be greater than *T_N_*, i.e., where *T_P_* is the total number of phages that have adsorbed and *T_N_* is the total number of bacteria that have become phage adsorbed. The value for these variables, as determined using Equations (A8) and (A12), fails however to adhere to that prediction. The reason for this is due to an assumption, in Appendix A.4.1, that phage titers are declining over time as a consequence of phage adsorptions, while bacterial densities remain constant. By contrast, in Appendix A.4.2, the assumption instead is that uninfected bacteria are declining in number over time while phage densities remain constant. Thus, the two equations are not modeling identical scenarios, with the failure to indicate reductions in phage titers over time in Equation (A12) predicting more losses of uninfected bacteria to phage adsorption (and therefore gains of infected bacteria) than would be the case were phages allowed to adsorb without replacement.

To address this discrepancy, we can calculate *T_N_* instead as a function only of those phages that have adsorbed over time, assuming losses of phages to adsorption, i.e., as via Equation (A8). To do this, we can substitute *T_P_*/*N*_0_ [7] for *kP*_0_*t* in Equation (A12). Thus,

(A13) $$T_{N}=N_{0}\left({1-e}^{-T_{P}/N_{0}}\right).$$

The reason this works is because *T_P_*/*N*_0_ is the ratio of adsorbed phages to adsorbable bacteria, also known as an actual multiplicity of infection (MOI_actual_) [13]. Furthermore, based on Poisson distributions, ‘e’, the base of natural logarithms, raised to the opposite of MOI_actual_ is equal to the fraction of bacteria that remain unadsorbed given that extent of phage adsorptions, e.g., [7]. Subtracting this number from 1 yields the fraction of bacteria that *have* been phage adsorbed. This is then multiplied by the starting concentration of bacteria, *N*_0_, to indicate the total number of bacteria that have been phage adsorbed. Based on Equations (A8) and (A13), then *T_P_* > *T_N_* now holds:

(A14) $$P_{0}\left({1-e}^{-kN_{0}t}\right)>N_{0}\left({1-e}^{-T_{P}/N_{0}}\right),$$

where $T_{P}=P_{0}\left({1-e}^{-kN_{0}t}\right)$ from Equation (A8). Thus,

(A15) $$T_{P}>N_{0}\left({1-e}^{-T_{P}/N_{0}}\right).$$

Solving the inequality numerically, this can be shown to hold true, i.e., such that *T_P_* >*T_N_*, for *P*_0_ ranging from 10^1^ to 10^9^ phages/mL and *N*_0_ also ranging from 10^1^ to 10^9^ phages/mL.
